# Donor derived hematopoietic stem cell niche transplantation facilitates mixed chimerism mediated donor specific tolerance

**DOI:** 10.3389/fimmu.2023.1093302

**Published:** 2023-02-16

**Authors:** Wensheng Zhang, Yong Wang, Fushun Zhong, Xinghuan Wang, Robert Sucher, Cheng-Hung Lin, Gerald Brandacher, Mario G. Solari, Vijay S. Gorantla, Xin Xiao Zheng

**Affiliations:** ^1^ Department of Plastic Surgery, University of Pittsburgh School of Medicine, Pittsburgh, PA, United States; ^2^ Thomas E. Starzl Transplantation Institute, University of Pittsburgh School of Medicine, Pittsburgh, PA, United States; ^3^ Transplantation Medical Center, Zhongnan Hospital of Wuhan University, Wuhan, Hubei, China; ^4^ Department of Visceral, Transplant, Thoracic and Vascular Surgery, University Hospital Leipzig, Leipzig, Germany; ^5^ Center for Vascularized Composite Allotransplantation, Department of Plastic and Reconstructive Surgery, Chang Gung Memorial Hospital, Chang Gung University College of Medicine, Tao-Yuan, Taiwan; ^6^ Department of Plastic and Reconstructive Surgery, Vascularized Composite Allotransplantation Laboratory, Johns Hopkins University School of Medicine, Baltimore, MD, United States; ^7^ Departments of Surgery, Ophthalmology and Bioengineering, Institute for Regenerative Medicine, Wake Forest School of Medicine, Winston-Salem, NC, United States

**Keywords:** tolerance, vascularized composite allotransplantation, mixed chimerism, bone marrow transplantation, hematopoietic stem cell niche, thymic central deletion

## Abstract

Compelling experimental evidence confirms that the robustness and longevity of mixed chimerism (MC) relies on the persistence and availability of donor-derived hematopoietic stem cell (HSC) niches in recipients. Based on our prior work in rodent vascularized composite allotransplantation (VCA) models, we hypothesize that the vascularized bone components in VCA bearing donor HSC niches, thus may provide a unique biologic opportunity to facilitate stable MC and transplant tolerance. In this study, by utilizing a series of rodent VCA models we demonstrated that donor HSC niches in the vascularized bone facilitate persistent multilineage hematopoietic chimerism in transplant recipients and promote donor-specific tolerance without harsh myeloablation. In addition, the transplanted donor HSC niches in VCA facilitated the donor HSC niches seeding to the recipient bone marrow compartment and contributed to the maintenance and homeostasis of stable MC. Moreover, this study provided evidences that chimeric thymus plays a role in MC-mediated transplant tolerance through a mechanism of thymic central deletion. Mechanistic insights from our study could lead to the use of vascularized donor bone with pre-engrafted HSC niches as a safe, complementary strategy to induce robust and stable MC-mediated tolerance in VCA or solid organ transplantation recipients.

## Introduction

The establishment of mixed chimerism (MC), as a result of donor-specific bone marrow transplantation, is one of the few approaches that have been reliably associated with “permanent,” rejection-free solid organ allograft acceptance in different species, notably in human renal transplantation (1–4). However, the concerns of life-threatening toxicities of conventional myeloablative conditioning procedures have prevented the widespread clinical application of these approaches. Efforts have therefore been undertaken to develop MC with non-myeloablative (reduced intensity) recipient conditioning and have been utilized to achieve immunological tolerance in rodent and few non-human primate models (5–15). Progressive peripheral clonal deletion of mature donor-reactive T cells has been demonstrated to be a key factor for the robust tolerance achieved by protocols of bone marrow transplantation with T-cell co-stimulation blockade (16–18). In contrast, the important contribution of central deletional mechanisms, arguably distinguishes MC regimens from other tolerance approaches (19–23). However, the key cellular mechanisms and role of the thymus in MC-mediated allotransplant tolerance remain ill-defined.

The longevity and multilineage feature of persisting donor leukocytes in organ allograft recipients suggest the presence of donor hematopoietic stem cells in organ allograft recipients and their proliferation/differentiation after transplantation (24, 25). Compelling evidence indicates that maintenance of hematopoietic stem cells (HSCs) and regulation of their self-renewal and differentiation *in vivo* depend on their specific microenvironment, which has been historically called the hematopoietic inductive microenvironment (26, 27) or ‘stem-cell niche’ (26). So far, most studies have focused on the bone marrow stem cell niche. Endosteal bone surfaces are lined with stromal cells. Spindle-shaped N-cadherin-expressing osteoblasts serve as niche cells to maintain quiescence and prevent differentiation of attached HSCs (28, 29). However, the presence and role of donor-derived osteoblastic stem cell niches in contributing to stable MC and donor-specific tolerance remain less well defined.

In the past two decades, successful vascularized composite allotransplantation (VCA) has highlighted its benefits for restoration of complex injuries or defects (30, 31). The unique features of vascularized bone component in VCA and their potential immunological contribution in facilitating stable MC and transplant tolerance have attracted academic and clinical attention (32, 33). In the present study, we utilized immunologically stringent VCA models to define the roles of donor bone marrow stem cell niches and the cellular mechanisms of thymic T cell central deletion in MC-mediated transplant tolerance.

## Materials and methods

### Animals

Male BN (RT1A^c^), LEW (RT1A^l^), Wistar-Furth (RT1A^u^) rats were purchase from Harlan Laboratories (Indianapolis, IN). The green fluorescent protein (gfp) transgenic SD rats were provided by Dr. Noriko Murase (University of Pittsburgh). Male B6 (H2^b^), B10.A (H2^k^), BALB/c (H2^d^), B6 nude (B6.Cg-*Foxn1^nu^*/J), BALB/c nude (CBy.Cg-*Foxn1^nu^*/J), B6.SCID (B6.CB17-Prkdc^scid^/SzJ), B6.Rag1 (B6.129S7-Rag1^tm1Mom^/J) mice were purchased from The Jackson Laboratories (Bar Harbor, ME). All animals were maintained under specific pathogen-free condition and procedures were performed according to the guidelines of the Institutional Animal Care and Use Committee at the University of Pittsburgh.

### Animal model of transplantation

VCA in a serial of MHC-mismatched rats (weighing 200-300g) and mice (weighing 25-30g) were performed as previously described (34–37). 1) vascularized skin/muscle (VSM) allograft without a bone component (myocutaneous flap supplied by the femoral vessels); 2) VSM allograft with donor bone marrow cells (BMC) transfusion (50x10^6^ cells equivalent of femur BMC (36), injected *via* tail vein); 3) skin/muscle/bone (VSMB) allograft (osteomyocutaneous flap containing distal ½ of the femur bone with adjacent muscle and skin supplied by the femoral vessels); 4) hind-limb allograft (containing ½ of the distal femur bone and rest of the hind-limb). For secondary skin grafting, full-thickness skin grafts were transplanted onto the dorsum of recipients (38, 39). For thymus transplantation, one section of the thymic lobe (size 3 x 5 mm) from tolerant or naïve B6 mice was transplanted underneath the kidney capsule of B6 nude mice as described previously (40, 41). We designed a novel VCA mouse model to directly test the roles of donor bone marrow HSC niche of VCA in facilitating stable MC in which hind limb allografts from athymic nude mice donor were transplanted to allogeneic SCID mice. We designed a unique thymus transplant mouse model to directly test the role of the chimeric thymus in MC-mediated transplant tolerance in which the chimeric thymus from tolerant VCA B6 recipients were transplanted into syngeneic B6.nude mice recipients, while the thymus from naïve B6 mice were used as control.

### Immunosuppressive treatment

Rat VCA recipients were treated as follows: 0.5ml anti-rat lymphocytes serum (ALS), intraperitoneal injection (i.p.) on postoperative day (POD) -4 and 0, cyclosporine A (0.6mg/kg/day i.p.) for the first 7 days after transplantation, followed by rapamycin (0.5 mg/kg/day, i.p.) for 14 days. Mouse VCA recipients were treated with co-stimulation blockade plus rapamycin protocol based on our previous study, in which we demonstrated a stable MC and skin allograft tolerance could be established across MHC barriers by a noncytotoxic, irradiation-free approach using costimulation blockade plus rapamycin treatment and high dose (2 x 10^8^) BMT (11).

### Evaluation of graft rejection

The transplanted allografts were inspected daily for clinical signs of rejection. Graft rejection was defined as epidermolysis/desquamation of the donor skin. Allografts displaying a healthy skin component with normal hair growth > 120 days post transplantation were considered signs of graft acceptance (14, 42). Histological evaluation was performed on skin and muscles sections stained with hematoxylin and eosin (H&E) to confirm the clinical observation.

### Flow cytometric analysis

Fluorochrome-conjugated antibodies against rat RT1Ac, CD45, CD45RA, CD3, CD4, CD8, CD11b/c, NK1.1, and mouse H2D^d^, CD19, CD3, CD4, CD8, CD11b, CD11c, NK1.1, Ter119, B220, TCRγδ, IgM, IgG, Vβ5.1/5.2, Vβ8, and Vβ11 for flow cytometry were purchased from AbD Serotec (Raleigh, NC), BD Pharmingen (San Diego, CA), and eBioscience (San Diego, CA). At the time points noted after transplantation, cells harvested from host rats or mice were analyzed using LSRII flow cytometer (BD Biosciences, San Diego, CA) and FlowJo software (Tree Star, Ashland, OR). For analysis of multilineage chimerism, transplant recipients were assessed serially for the presence of donor hemopoietic cell lineages in peripheral blood (PB), bone marrow, thymus, lymph nodes, and spleen by detecting anti-rat MHC-class I antibody (RT1Ac) or gfp, and linage-specific cell surface markers. For analysis of T cell receptor (TCR) Vβ families, PB was obtained from B6 recipients that received B10.A hind-limb allografts at different time points after transplantation. The proportion of CD4^+^ T cells expressing each Vβ was determined (11). For detection of anti-donor antibodies, serum collected from B6 recipients was incubated with splenocytes of donor B10.A mice. Splenocytes were then stained with antibodies and assessed for the levels of donor-specific IgM or IgG antibodies on CD4^+^ T cells.

### Adoptive cell transfer

Single-cell suspensions were prepared from spleens and lymph nodes of tolerant mixed chimeric or naïve B6 mice. After T-cell enrichment and subsequent deletion of CD25^+^, Ter119^+^, B220^+^, CD8^+^, CD11b^+^, TCRγδ^+^, and NK1.1^+^ cells with magnetic beads, 5x10^6^ CD4^+^CD25^-^ T cells were adoptively transferred into B6.Rag1^-/-^ mice *via* tail vein injection (11).

### Histologic studies

The skin, muscle, femur, tibia, and thymus were harvested from the recipients or allografts on determined days following transplantation and processed for paraffin sections. For morphologic evaluation, sections were stained with H&E. For immunofluorescence imaging, sections were cut at 6 µm and stained with primary antibodies: anti-Osterix, anti-c-Kit, anti-CK5, anti-CK8, anti-CD11c from Abcam (Cambridge, MA) or anti-DEC205 from Santa Cruz (Dallas, TX), followed by fluorescent secondary antibodies. DAPI (Sigma-Aldrich) was used to stain the nucleus. The slides were evaluated in a blinded fashion.

### Statistics

Values are expressed as mean ± SEM. Differences between experimental groups were analyzed by Student’s *t*-test for two groups or by one-way ANOVA test for multiple comparisons. The allograft survival times between groups were calculated and compared using a Logrank test. ‘*p’* values < 0.05 were considered significant for all statistical tests.

## Results

### The vascularized bone component in VCA promotes allograft engraftment and tolerance

**Figure 1:**
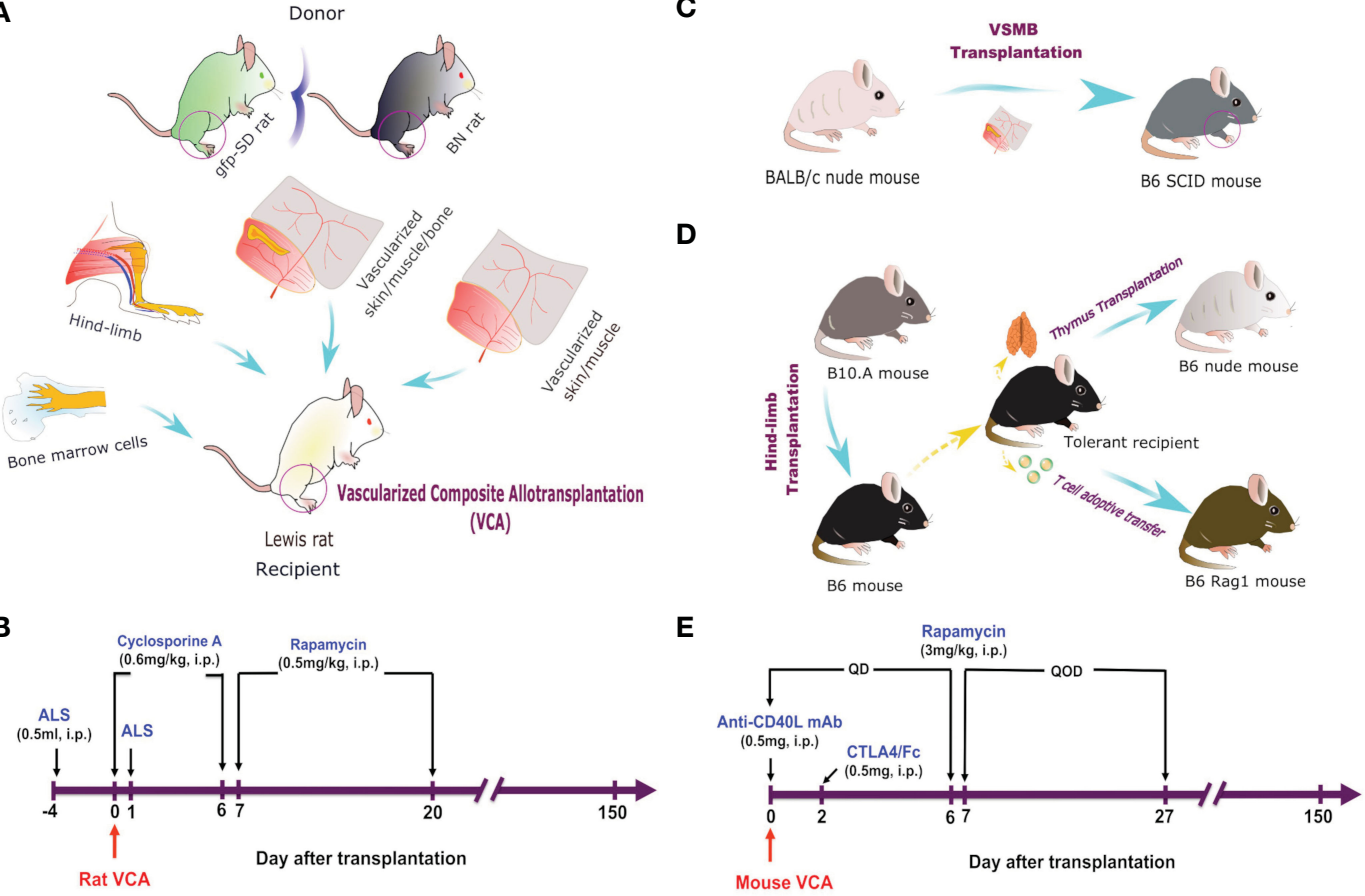
VCA models and immunosuppressive regimes. **(A)** Hind-limb, VSMB, VSM, and BMC (50 x 10^6^) transplantation from either BN or gfp-SD donor rats to LEW rat recipients. **(B)** Recipient rats were sequentially treated i.p. with ALS, cyclosporine A, and rapamycin for 21 days. **(C)** VSMB or VSM transplantation from BALB/c nude mice to B6 SCID mice. **(D)** Hindlimb transplantation from B10.A mice to B6 mice. Thymus transplantation and T cell adoptive transfer were performed from tolerant recipients to B6 nude mice. **(E)** VCA recipient mice were treated i.p. with anti-CD40L mAb, CTLA4/Fc, and rapamycin for 28 days.

**Figure 2:**
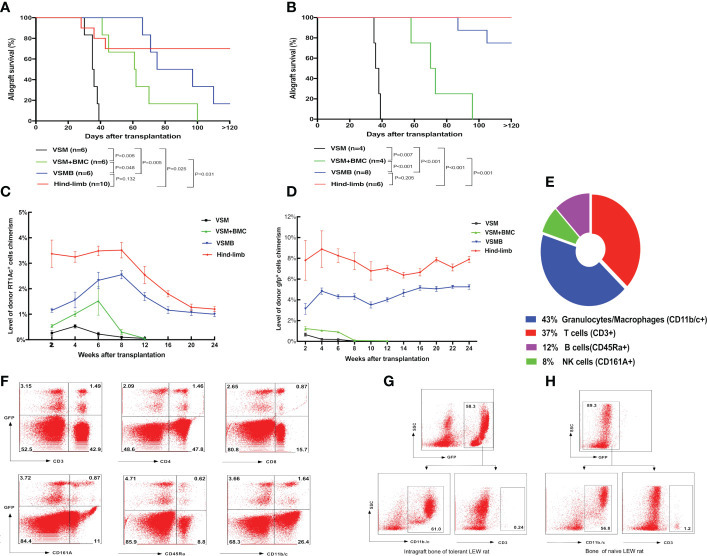
**(A, B)** Allograft survival in different VCA transplant models under the same immunosuppressive regimen. LEW recipients were transplanted with VSM, VSM+BMC, VSMB or hind-limb allografts from BN donors **(A)** or gfp-SD donors **(B)** and treated with the same sequential immunosuppressive strategy. Logrank analysis of the survival curves indicated statistical significance (p< 0.05) for VSM group vs other three groups, VSM+BMC group vs VSMB or hind-limb group, respectively. **(C, D)** Longitudinal changes of donor cell percentage in peripheral blood leukocytes of VCA recipients. In BN to LEW **(C)** and gfp-SD to LEW **(D)** transplantation models, donor cell chimerism persists for over 24 weeks in the peripheral blood of both VSMB and hindlimb allograft recipients. In contrast, only transient, lower-level of donor cells can be detected in either VSMB+BMC or VSM recipients during 2-6 weeks post transplantation, which then gradually disappeared at 8-12 weeks post-transplantation. The percentage of RT1Ac^+^ or gfp^+^ cells in peripheral blood is analyzed by flow cytometry, gating of all leukocytes using forward versus side scatter (FSC vs SSC) plot by including all leukocyte subpopulations (lymphocytes, monocytes, granulocytes) and excluding debris/dead cells, followed by analysis of donor MHC-I (RT1Ac) or gfp^+^ expression. For levels of multi lineage donor chimerism, lineage-specific cell markers were further assessed to identify subpopulations of leukocytes. **(E, F)** Multilineage chimerism detected in recipients the received VSMB allografts from the gfp-SD rat donors. **(E)** Mean percentage of chimeric subpopulations in gfp^+^ PBL of VSMB rat recipient analyzed by flow cytometry at 6 weeks post-transplantation (n=8). **(F)** Flow cytometric analysis of PBL of the chimeric VSMB recipients at 24 weeks post-transplantation (n=8). Representative dot plots show that around 5% of PBL were donor-derived gfp^+^ cells with multilineage subpopulations: T cells (CD3^+^, 32% of gfp^+^), CD4-T cells, CD8-T cells, NK cells (CD161A^+^, 18% of gfp^+^), B cells (CD45Ra^+^, 11.6% of gfp^+^), granulocytes/macrophages (CD11b/c^+^, 31% of gfp^+^). **(G, H)** Donor vascularized bone components in VSMB recipients maintain viable BMC for long periods of time post transplantation. Flow cytometric analysis on BMC of bone graft extracted from LEW recipients at 20 weeks after transplantation of VSMB from donor gfp-SD rats (n=6) and typical BMC phenotypes (CD11b/c^+^, CD3^-^) were identified by gating on gfp^+^ leukocytes **(G)**. The similar phenotypes were exhibited on BMC from the naïve LEW rats by gating on gfp- leukocytes **(H)**.

In BN to LEW rat VCA, with the same immunosuppressive treatment (**Figures 1A, B**), the VSM recipients (n = 6) rejected allografts with a median survival time (MST) of 39 days and the VSM plus BMC recipients (n= 6) had delayed allograft rejection (MST=70 ± 10 days, p= 0.005). In contrast, prolonged allografts survival was demonstrated in both VSMB (MST=100 ± 12 days, n= 6, p= 0.005) and hind-limb (MST >120 days, n= 10, p= 0.025) recipients (**Figure 2A**). Similar results were observed in gfp-SD to LEW transplants, in which both VSMB (n=8) and hind-limb (n=6) allografts survived (both MST >120 days) significantly longer than the groups of VSM (MST=47 days, n=4) and VSM+BMC (MST=71.5 days, n=4, p<0.001) (**Figure 2B**). Long-term surviving recipients were challenged with secondary skin allografts (n=6) and accepted the donor-type skin graft, but rejected third-party allografts, indicating a donor-specific tolerance.

### The vascularized bone component bearing donor bone marrow HSC niches promotes stable hematopoietic MC

The donor hematopoietic cells in PB of recipients were dynamically evaluated by flow cytometry. In BN to LEW VCA, either VSM or VSM+BMC recipients only exhibited transient, lower-level donor RT1Ac^+^ cell chimerism, which decreased rapidly by 2-6 weeks post transplantation and correlated with the signs of allograft rejection, while VSMB and hind-limb recipients maintained stable MC (donor cells account for 2% of leukocytes) up to 24 weeks post transplantation (**Figure 2C**). Similarly, in gfp-SD to LEW VCA, donor gfp^+^ cell chimerism (4-6%) persisted for over 24 weeks in the PB of both VSMB and hind-limb recipients, while only 0.5-1% of donor gfp^+^ cells were detected in either VSM or VSMB+BMC recipients during the 2-8 weeks post transplantation and disappeared thereafter (**Figure 2D**). Moreover, multilineage chimerism was evidenced by the consistent gfp^+^ cell profile (percentage of chimeric subpopulations) in PB at 6 weeks (**Figure 2E**) and 24 weeks (**Figure 2F**) post- transplantation in durable chimeric VSMB recipients. The bone marrow from the excised donor bone grafts at 20 weeks post transplantation maintained 40%-60% gfp^+^ cells with typical BMC phenotype (**Figures 2G, H**).

### Donor-derived HSCs home to the recipient bone marrow compartment and lymphoid organs after VCA

**Figure 3:**
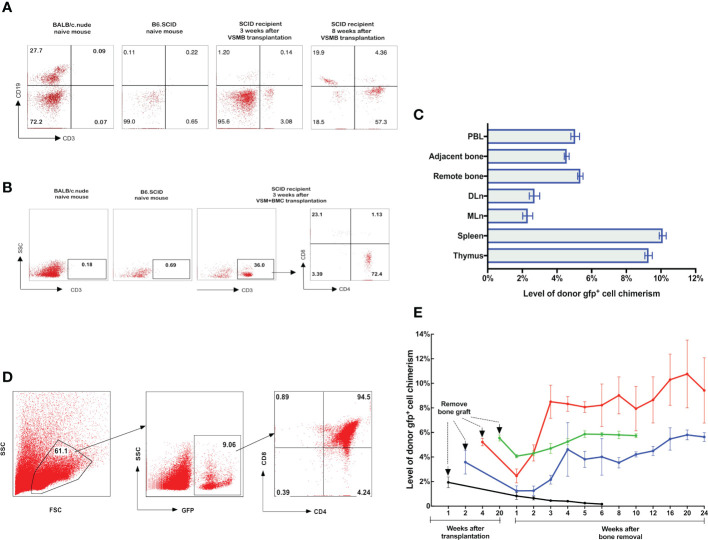
**(A, B)** Detection of donor progenitor cells differentiation in SCID mice after receiving VSMB or VSM+BMC from nude mice. VSMB or VSM+BMC (10 x 10^6^) from nude mice were transplanted to SCID mice. CD3^+^, CD4^+^, CD8^+^, and CD19^+^ lymphocyte sub-populations in blood of SCID mice were assessed by flow cytometry after transplantation. The parent population is lymphocyte, which was gated by forward versus side scatter (FSC vs SSC) plot, followed by analysis of lymphocyte subsets (T and B cells) using CD3 vs CD19 (Fig 3A) or CD3 vs SSC, CD4 vs CD8 (Fig 3B) plots. **(A)** CD3^+^ T cells were detected and steadily increased in PBL of SCID recipient at 3 weeks and 8 weeks after VSMB transplantation (n=4). **(B)** Mature CD3^+^ T cells were detected in PBL of SCID recipient only at 3 weeks after VSM+BMC. Data are representative of four independent experiments. **(C, D)** Tracking of donor cells homing in VSMB allograft recipient with sustained stable MC. **(C)** At 20 weeks post VSMB transplantation from gfp-SD to LEW, the gfp^+^ cells in PB, DLn, MLn, splenocytes, thymocytes, bone marrow of the tibia adjacent to allograft (adjacent bone) and contralateral tibia (remote bone) of recipients were assessed by flow cytometry. FSC vs SSC plots were used to firstly remove cellular debris and dead cells, and gfp^+^ subpopulations were then measured for its percentage in the live cells. Data show the results (mean ± SEM) of four recipients. **(D)** Representative flow cytometric analysis on thymocytes of VSMB allograft recipient at 20-week post-transplantation. Around 9% of thymocytes are donor gfp^+^ cells, in which gfp^+^CD4^+^CD8^+^ cells account for >90%. **(E)** The influence of vascularized bone components in inducing and maintaining MC. LEW rats received VSMB allografts from gfp-SD rats and the bone component of VSMB graft was removed from the recipients at 1, 2, 4, and 20 weeks after transplantation. The gfp^+^ cells in PBL were sequentially assessed by flow cytometry. The level of donor chimerism experienced a transient decrease, then increased to around 6.0-12.0%, and persisted for > 24 weeks after the bone component being removed at 2, 4, 20 weeks, except that at 1 week. Data points represent results (mean ± SEM) for recipients at each time point: week 1 (n=2), week 2 (n=6), week 4 (n=6), week 20 (n=8).

We tracked the homing of donor-derived cells in gfp-SD to LEW VCA recipients with sustained MC using flow cytometry. At 20 weeks after VSMB transplantation, donor gfp^+^ multilineage cells were evident in the draining lymph nodes (DLn, 3.5%), mesenteric lymph nodes (MLn, 2.5%), spleen (11%), thymus (9%), and bone marrow from adjacent bone marrow (5%) as well as remote bone marrow (5.5%) (**Figure 3C**). In addition, most of gfp^+^ cells (94%) in thymus were CD4^+^CD8^+^ cells, a transition status of T-cell differentiation during its maturation, demonstrating the undergoing maturation of donor T cell progenitors in recipient thymus (**Figure 3D**).

To test that the donor bone marrow HSC niche of VCA allograft plays a role in establishing a stable MC, we employed a novel VCA mouse model (43), in which B6.SCID mice received either VSMB or VSM+BMC from BALB/c nude mice without immunosuppressive treatment (**Figure 1C**). Flow cytometric data showed that CD3^+^ T-cells (3% of leukocytes) were detected in PB of B6.SCID recipients with VSMB at 3 weeks and increased to 50% at 8 weeks post transplantation (n=4) (**Figure 3A**). In contrast, CD3^+^ T-cells in PB of VSM+BMC recipients (n=4) were only detected at 3 weeks, but not 8 weeks, post transplantation (**Figure 3B**). Moreover, B6.SCID recipients with VSMB (n=4) were capable of rejecting skin allografts from the third-party (B10.A) mice after 4 weeks of transplantation, while B10.A skin grafts did not elicit rejection in VSM+BMC recipients (data not shown).

### The donor bone component in VCA is essential for establishment but not maintenance of stable MC

To assess whether the donor bone component of VCA is imperative for maintenance of stable MC, we removed the bone component from recipients that received VSMB of gfp-SD rats at 1, 2, 4, 20 weeks after transplantation. The remaining skin and muscle tissue were reconstructed as vascularized myocutaneous allografts. As shown in **Figure 3E**, with bone removal at 1 week, the gfp^+^ cells in PB of recipients decreased rapidly, leading to rejection of the remaining VSM. In contrast, after the bone component was removed at 2, 4, and 20 weeks, the gfp^+^ cells in PB transiently decreased to 1-2% (of leukocytes), then resumed quickly to 6.0-12.0% and persisted at 4.5-5.5% for > 24 weeks (60% of recipients). In some recipients (30%), the donor cell chimerism reached 90%. No chimeric recipients demonstrated signs of graft versus host disease (GVHD). All remaining skin/muscle allografts maintained their engraftment permanently (>150 days) and the tolerance was confirmed by acceptance of secondary donor skin grafts (data not shown).

**Figure 4:**
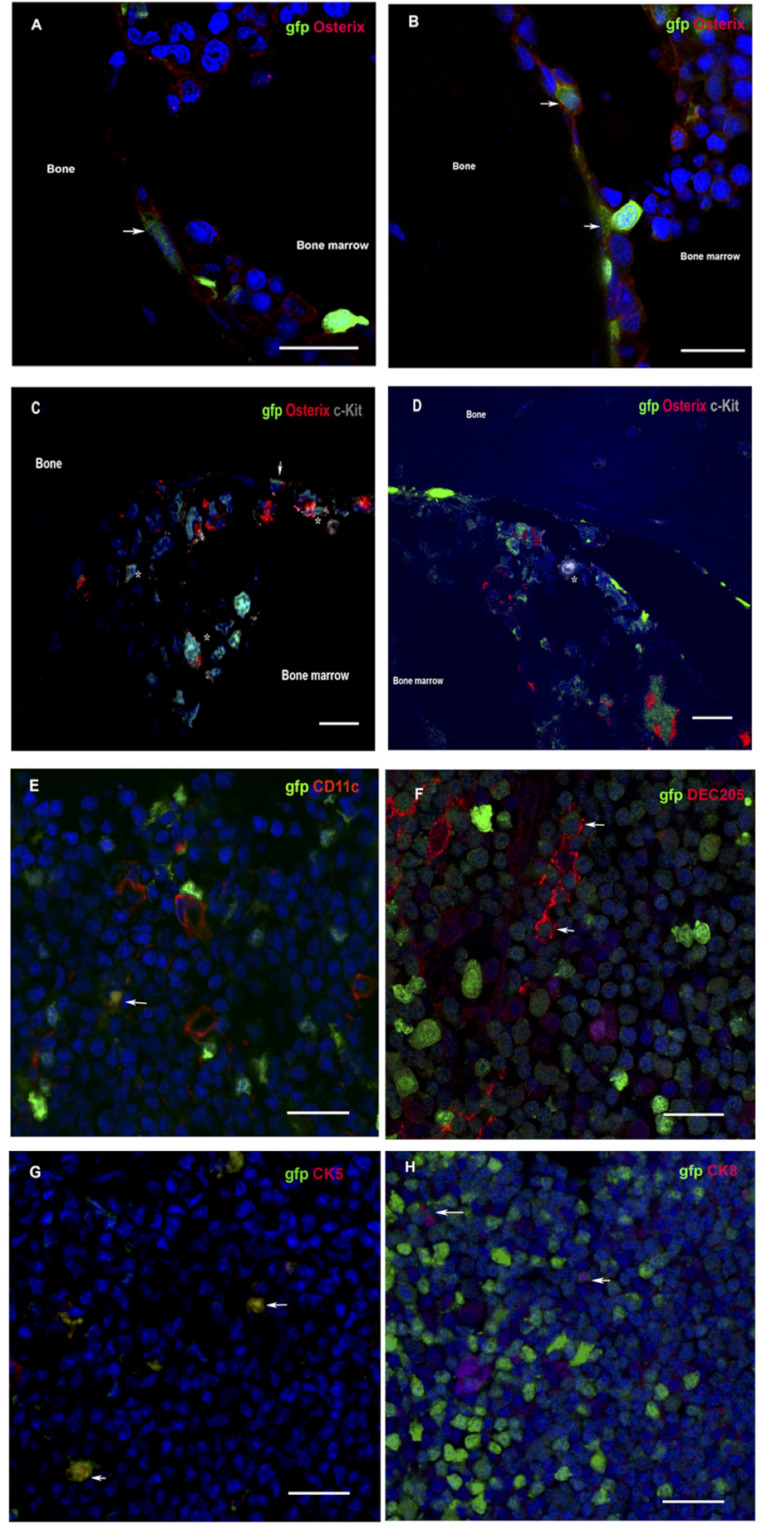
**(A–D)** Donor osteoblasts and HSCs home to chimera recipient bone after VCA. Immunofluorescence of bone sections from the contralateral femur of recipients at 20 weeks after transplantation of VSMB from gfp-SD to LEW rats. **(A, B)** Donor osteoblasts (arrow) were observed in the endosteal area as revealed by co-staining for gfp^+^ (green) and osterix^+^ (red). (1200X). **(C, D)** Donor HSCs (asterisk) were also detected, near osteoblasts (arrow), in endosteal and bone marrow area by co-staining of gfp+ cells with c-kit+ (grey) (600X). **(E–H)** Donor DC and TEC home to chimera recipient thymus after VCA. Immunofluorescence of thymus sections from recipients at 20 weeks after transplantation of VSMB from gfp-SD to LEW rats (400X). **(E–H)** Donor DC and TEC (arrow) are observed in area around corticomedullary junction as revealed by co-staining of gfp^+^ (green) cells with DC (CD11c^+^ and DEC205^+^, red) and TEC (CK5^+^ and CK8^+^, red) markers. Nuclei were stained with 4’,6’- diamidino-2-phenylindole (DAPI, blue). Scale bars represent 25μm. The representative sections are from four independent experiments.

To evidence donor-derived HSC niches seeding in the recipient bone compartment, the femoral component of gfp-VSMB recipients harvested at 20 weeks post transplantation were immunostained for osteoblasts and HSCs markers (anti-osterix and anti-c-kit). In agreement with previous studies (33, 44–47), donor osteoblasts were found localized in endosteal surfaces as revealed by co-staining for gfp^+^ and osterix^+^, and donor HSCs were also detected by co-staining of gfp^+^ cells with c-kit^+^ near osteoblasts (**Figures 4A–D**).

### Thymic central deletion play a key role in MC-mediated VCA tolerance

Since both dendritic cells (DC) and thymic epithelia cells (TEC) have been found to critically contribute to physiological self-tolerance by mediating T cell central deletion (48–51), we sought to determining whether donor-derived DC and TEC constitute the MC in the thymus of tolerant VCA recipients. Indeed, the immuno-fluorescence staining on the thymus sections of tolerant VSMB recipients showed that both donor-derived DC and donor-derived TEC were present in thymic cortico-medullary junction field, as evidenced by co-staining of gfp^+^ cells with CD11c^+^ and DEC205^+^ (DC markers) as well as cytokeratin (CK)5^+^ and CK8^+^ (TEC markers) cells (**Figures 4E–H**).

**Figure 5:**
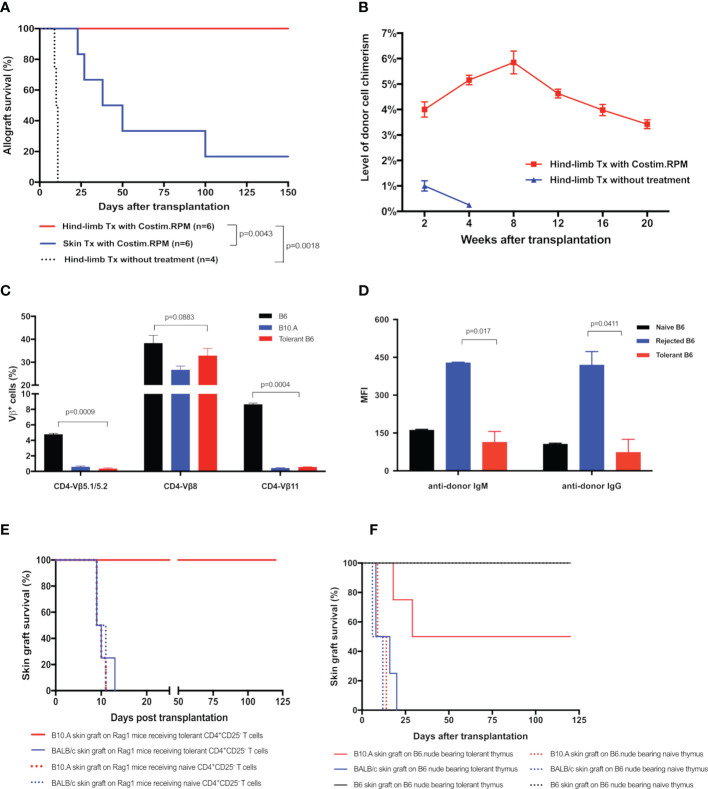
**(A)** Survival of hind-limb or skin allografts in mice transplant model. B6 mice received hind-limb allografts from B10.A donors and were treated with a short-term co-stimulation blockade plus rapamycin protocol. Full-thickness skin allograft recipients with the same immunosuppressive treatment and hind-limb allograft recipients without treatment were served as controls. **(B)** Donor cell percentage in PBL of B10.A to B6 hind-limb allograft recipients. The percentages of H2D^d+^ cells in PBL of recipients were analyzed by flow cytometry. **(C, D)** The deletion of donor-specific TCR Vβ clones and the absence of donor-specific antibodies in tolerant recipients. **(C)** TCR Vβ expression profiles in PBL of the tolerant recipients were analyzed at POD 120 by flow cytometry and expressed as the percentages of TCR Vβ5.1/5.2^+^, Vβ8^+^, Vβ11^+^ cells in CD4^+^ cells The Naïve B6 and naïve B10.A mice were used as controls. **(D)** Donor-specific IgM, IgG antibodies in PBL of recipients were measured at POD 120. Splenocytes from the donor B10.A mouse were first incubated with sera collected from tolerant recipients, or recipients that underwent graft rejection, or naïve B6 mice, and then stained with fluorochrome-conjugated anti-mouse IgM, or IgG, and CD4 antibodies. Splenocytes were analyzed by flow cytometry for anti-donor antibodies expression on CD4^+^ T cells. Results were displayed as mean fluorescence intensity (MFI) ± SEM. **(E)** Survival of skin allografts in Rag1^-/-^ mice receiving CD4^+^CD25^-^ T cells. CD4^+^CD25^-^ T cells (5x10^6^) harvested from the splenocytes of tolerant B6 mice were i.v. injected to the B6.Rag1^-/-^ mice. Naïve B6 CD4^+^CD25^-^ T cells were used as controls in adoptive transfer. Recipient Rag1^-/-^ mice were further challenged with skin allografts from B10.A (donor), BALB/c (third party), and naïve B6 mice (native control). Skin grafts from naïve B6 mice were accepted by all adoptive transferred B6.Rag1^-/-^ mice recipients. **(F)** Survival of skin allografts in nude mice receiving thymus transplant. Thymus lobes from the tolerant B10.A-B6 chimeric mice were transplanted into the subrenal capsule of the B6 nude mice. At POD14, skin allografts from B10.A (donor), BALB/c (third party), and naïve B6 mice were transplanted simultaneously to the same thymus-bearing B6 nude mice.

To determine the critical role of thymic central deletion of alloreactive T cells in MC-mediated VCA tolerance, an MHC-mismatched B10.A to B6 mouse hind-limb transplantation model was utilized and recipients were treated with our established co-stimulation blockade plus rapamycin protocol (11) (**Figures 1D, E**). The results showed that all treated hind-limb recipients (n=6) accepted B10.A allografts with persist peripheral blood MC (3-6% donor cells) for >20 weeks post transplantation. In contrast, both treated skin allograft recipients (n=6) and untreated hind-limb allograft recipients (n=4) rejected the allografts at MST of 44 days and 10.5 days, respectively. Only very low levels (<1%) of MC were transiently detected in untreated hind-limb recipients during the 1-3 weeks post transplantation (**Figures 5A, B**). Donor-specific tolerance of long-term survival (>100 days) recipients was confirmed by acceptance of the donor (B10.A) skin grafts and rejection of third-party (BALB/c) skin allografts, which were confirmed by both inspection of clinical sign of rejection and histological assessments of skin samples. The H&E-stained skin sections of clinically accepted secondary donor skin allografts showed no or rare inflammatory or immune cell infiltrates, and was diagnosed as Banff grade 0. In addition, donor-antigen specific Vβ5.1/5.2^+^ and Vβ11^+^, but not irrelevant Vβ8^+^, T cells were significantly reduced in PB of tolerant MC recipients at POD 120 after transplantation (n=6, p< 0.05), as compared with naïve B6 controls (**Figure 5C**). Furthermore, donor-specific IgM and IgG were remained at basal levels or lower in tolerant VCA recipients, but increased significantly in rejected VCA recipients (**Figure 5D**).

To confirm the donor antigen-specific T cells were deleted in tolerant VCA recipients, we adoptively transferred CD4^+^CD25^-^ T cells from the limb-tolerant or naïve B6 mice into immunoincompetent B6.Rag1^-/-^ mice, then challenged recipients with skin grafting 1 day after the adoptive transfer. As shown in **Figure 5E**, B6.Rag1^-/-^ recipients receiving naïve CD4^+^CD25^-^ T cells triggered rapid rejection of both B10.A (MST=9.5 days) and BALB/c (MST=11 days) skin grafts. In contrast, B6.Rag1^-/-^ mice receiving tolerant CD4^+^CD25^-^ T cells accepted B10.A grafts (MST >120 days) and rejected BALB/c skin grafts (MST=10 days). Moreover, throughout a follow-up >12 weeks period after skin transplantation, partial deletion of Vβ5.1/5.2^+^ and Vβ11^+^ CD4 T cells was observed in Rag1^-/-^ mice receiving tolerant CD4^+^CD25^-^ T cells (**Supplementary Figure 1**). These results provide additional evidence for clonal deletion of donor-reactive CD4 T cells in VCA tolerance.

To ascertain the role of the thymus in MC-mediated VCA tolerance, we employed a unique thymus transplant model, in which thymus lobes from the B10.A to B6 VCA tolerant recipients (30 weeks old) or age-matched naïve B6 mice were transplanted underneath the kidney capsule of the B6.nude mice (**Figure 1D**). Two weeks later, B6.nude recipients were challenged with secondary skin grafting. As shown in **Figure 5F**, the nude mice bearing naïve thymus rejected both B10.A (donor-specific) and BALB/c (third-party) skin grafts at an MST of 11.5 and 9 days respectively. In contrast, nude mice bearing tolerant chimeric thymus showed a prolonged engraftment for donor-specific B10.A skin grafts (MST=64.5 days) with 50% of them surviving indefinitely (>120 days, p<0.05 vs naïve thymus-bearing recipients), while rejecting third-party BALB/c skin grafts (MST =11.5 days). Furthermore, Vβ5^+^ and Vβ11^+^ CD4 T cells were partially reduced in tolerant thymus-bearing nude mice during the 12-week follow-up period (**Supplementary Figure 1**), indicating that intrathymic clonal deletion plays a critical role in MC-mediated VCA tolerance.

## Discussion

In this study under the same non-myeloablative regimens we observed that the VCA with an intra-graft bone component do promote stable MC, long-term allograft survival and donor specific transplant tolerance. In contrast, VCA without a vascularized bone component, such as VSM, or even VSM combined with low dose donor BMC transfusion (equivalent of femur bone marrow cells), only exhibited transient, lower-level MC in periphery blood, and failed to prevent allograft acute rejection. Similar studies were reported by multiple groups using bone graft transplantation (43, 52, 53) or vascularized bone marrow transplantation (33, 36, 37) to induce hematopoietic chimerism and allograft tolerance.

Moreover, multilineage chimerism is evidenced by the similar gfp+ cell profile in peripheral blood in comparison with gfp- cell profile in durable chimeric recipients and the fact of most of gfp^+^ cells (94%) in thymus are CD4^+^CD8^+^ double positive cells, a transition status of T cell differentiation during its maturation, indicate the homing, proliferating, differentiating and maturating of donor derived HSC in the recipients. Furthermore, at 20 weeks post transplantation, the bone marrow from the excised donor bone grafts still contained 40% - 60% gfp^+^ cells with typical bone marrow cell phenotype, supporting the hypothesis that the vascularized bone component of VCA bears HSC niches to facilitate a stable MC.

We designed a novel VCA mouse model, in which hind limb allografts from athymic nude mice donor were transplanted to allogeneic SCID mice to directly test the roles of donor bone marrow HSC niche of VCA in facilitating stable MC. Nude mice have a spontaneous FOXN1 gene deletion resulted in a non-functional thymic cortical defect – athymic, but have functional bone marrow hematopoietic progenitors (54, 55); while SCID mice have V(D)J recombinant defect preventing genetic defect T progenitor from maturation, but have a functional thymus (56). Since mature T cells do not develop in either strain, any mature functional T cells in SCID recipients receiving VSMB grafts from nude donors will be the result of the migration of nude donor hematopoietic progenitors from bone marrow of VSMB graft to the SCID recipient’s thymus, therein undergo differentiation and maturation. Since there were BMC preexisting in donor VSMB bone marrow, we used VSM plus 10 x10^6^ BMC from nude mice as control. Indeed, we found CD3^+^ T cells in the PB of SCID recipients receiving nude VCA at 3 weeks post nude VCA transplant, which steadily increased to 50% at 8 weeks post transplantation. Moreover, these VCA SCID recipients were capable of rejecting skin allograft after 4 weeks of transplantation, indicating the CD3^+^ T cells were functional active. In contrast, only transient T cells were detected in the PB of SCID recipients receiving VSM+BMC from nude mice, and they accepted skin allograft after VSM transplantation. These results provided convincing evidences that the donor bone marrow HSC niche of VCA plays a role in establishing a stable MC after transplantation.

We further sough to determine the roles of the donor bone marrow HSC niche of VCA in maintaining a stable MC. Interestingly we found that only two weeks were required for the donor vascularized bone component of VCA to establishing a MC in this rodent VCA model. In this study donor-derived osteoblasts and HSC were detected homing closely to the endosteal region in trabecular bone of chimeric VSMB recipient by immunofluorescence staining, which are in line with the features ascribed to endosteal HSC niche (44–46, 57–59) suggesting that donor HSC niches were reconstituted in recipients’ bone marrow as a continuous renewable donor HSC source (53) and maintained stable MC. The observation of dynamic changes of the gfp^+^ cells in PB of VCA recipients after the bone component being removed at 2, 4, 20 weeks post VCA, transiently decreased, then resumed quickly and persisted at previous level for > 24 weeks (60% of recipients) suggested that donor HSC niches were integrated into the recipient immune system for maintaining the homeostasis of stable MC. It was surprising and fascinating result that the donor cells chimerism reach to 90% in 30% recipients after the bone component being removed at 4 weeks post VCA and without any sign of GVHD. The mechanisms and prospective of these results and the complex interplay between HSC niches in the bone allograft and recipient immune system merits further investigation.

In parallel with the development of stable MC, the donor-specific tolerance to composite tissues, including highly immunogenic skin allografts, was achieved in both mouse and rat bone-containing VCA models as the chimera accepted secondary donor skin allografts, but not third-party skin allografts without immunosuppression. The adoptive transfer of CD4^+^CD25^-^ effective T cells from tolerant VCA recipients into Rag1^-/-^ mice failed to reject donor strain skin allograft, but were capable to reject third party skin allografts suggesting the role of clonal deletion of alloreactive cells in the induction and maintenance of donor-specific tolerance in these VCA models. Furthermore, the immuno-fluorescence staining on the thymus sections of tolerant VSMB recipients shows that both donor-derived DC and donor-derived TEC are present in thymic cortico-medullary junction field, as evidenced by co-staining of gfp^+^ cells with CD11c^+^ and DEC205^+^ (DC markers) as well as CK5^+^ and CK8^+^ (TEC markers) cells. Since DC and TEC are essential in T cell thymic clonal deletion of self-tolerance (50, 60), our finding, in line with a prior similar study (61), strongly suggested that the presence of donor-derived DC and TEC in thymus of tolerant VCA recipients play an important role in the central deletion of alloreactive T cell clones.

We designed a unique thymus transplant mouse model to directly test the role of the chimeric thymus in MC-mediated transplant tolerance in which the chimeric thymus from tolerant VCA B6 recipients were transplanted into syngeneic B6.nude mice recipients, while the thymus from naïve B6 mice were used as control. Since nude mice are athymic and consequently lack T cells, any detectable functional CD3^+^ T cells in B6.nude recipients receiving chimeric thymus grafts from B6 tolerant donors will be the result of the migration, differentiation and maturation of nude HSC in the chimeric thymus. The nude mice bearing naïve-thymus acutely rejected both B10.A and BALB/c skin grafts. In contrast, the nude mice bearing tolerant MC thymus acutely rejected BALB/c skin grafts, while demonstrated a prolonged engraftment for donor-specific B10.A skin allografts with 50% of them survived infinitely. Since the frequency of donor-derived DC and/or TEC in the donor thymus was not defined and the peripheral tolerance could not be overlooked, this experiment alone is not sufficient to indicate the extent of the role of central deletion in VCA tolerance. Nevertheless, it is reasonable to speculate that the chimerism in the donor thymus is inducing central tolerance. In addition, by using Vβ5, Vβ11, and Vβ8 TCR probes we evidenced specific deletion of alloreactive T cell clones in peripheral blood of chimeric thymus recipients. These results provided favorable evidence in a hind-limb VCA model that the thymus plays an important role in MC mediated allograft tolerance through a mechanism of intrathymic central deletion of allo-reactive T cell populations.

This study supports the hypothesis that vascularized bone grafts bear donor HSC niches for sustained donor derived HSC/mesenchymal stem cells production, homing and renewal, provide a unique biologic opportunity to facilitate stable MC and transplant tolerance without harsh myeloablation.

**Figure 6:**
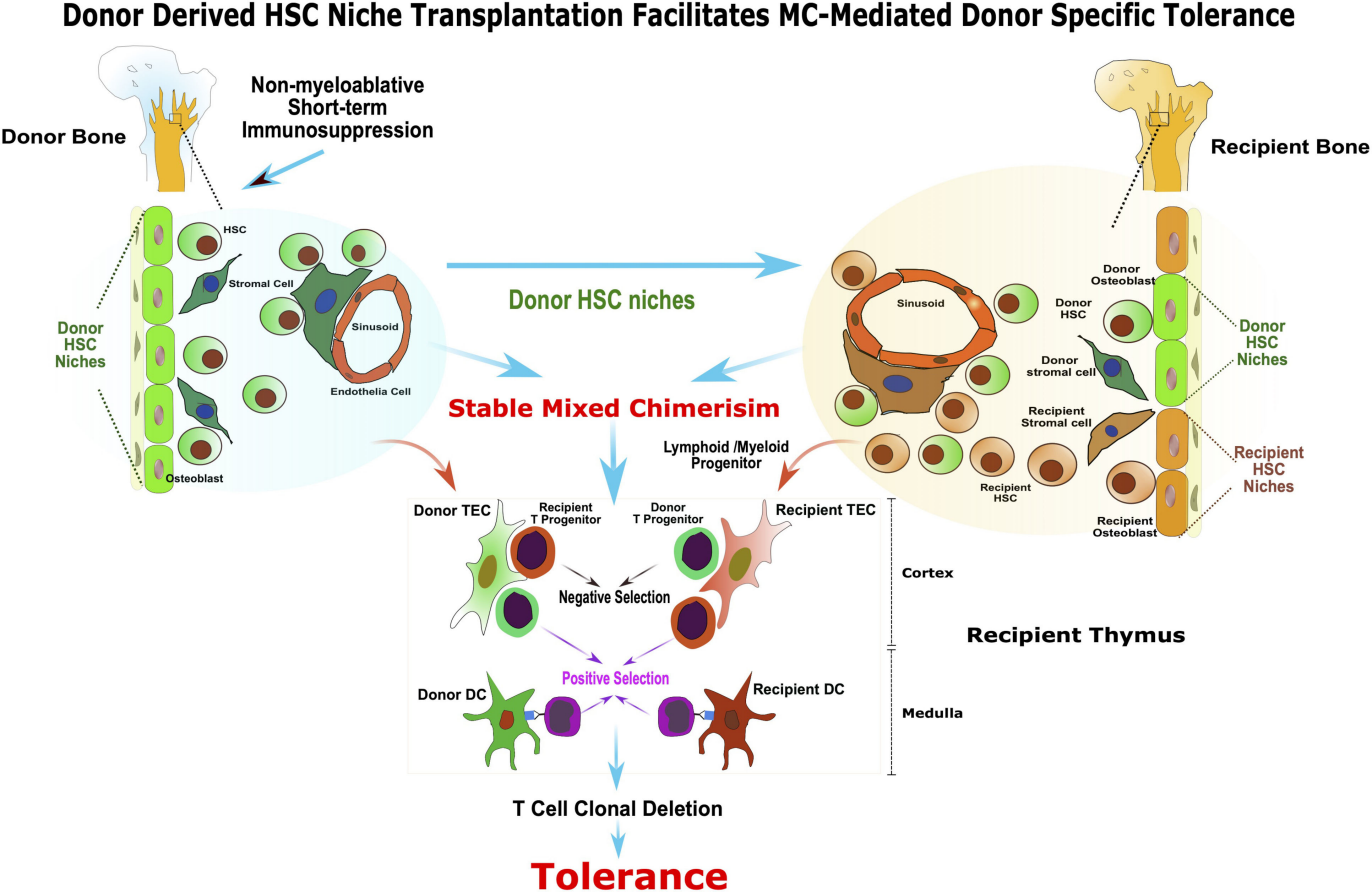
Donor derived HSC niche transplantation facilitates MC-mediated donor specific tolerance. The presence of functional donor derived HSC niche is required for the maintenance of stable MC. The vascularized bone graft bears donor HSC niche enabling sustained donor-derived HSC/mesenchymal stem cells production, homing and renewal, and facilitate stable MC and tolerance without harsh myeloablation. In addition, the transplanted donor HSC niche in VCA facilitated the donor HSC niche seeding to the recipient bone marrow compartment and contributed to the maintenance of homeostasis of stable MC. The co-existence of donor and recipient derived stromal cell and DC in recipient’s thymus as a result of stable MC may provide the cellular mechanisms for alloreactive T cell central deletion and allograft tolerance.

In summary, this study provides a mechanistic insight into the MC-mediated transplantation tolerance (**Figure 6**) and paves a way for implementation of a safe, complementary strategy with concomitant donor HSC niches transplant to facilitate stable hematopoietic MC and tolerance. The use of vascularized donor bone, such as femur, ilium or craniofacial allografts from human donors, in solid organ transplantation could be a clinically feasible approach for inducing robust immune tolerance, while reducing or eliminating the need for chronic immunosuppression.

## Data availability statement

The original contributions presented in the study are included in the article/**Supplementary Material**. Further inquiries can be directed to the corresponding author.

## Ethics statement

The animal study was reviewed and approved by Institutional Animal Care and Use Committee at the University of Pittsburgh.

## Author contributions

XZ and WZ designed experiments, analyzed data and interpreted results. WZ, YW, FZ, RS, and C-HL performed animal experiments, immunologic assays and data acquisition. WZ and XZ wrote the manuscript and prepared the figures. VG, GB, and MS provided critical scientific input and reviewed and edited the manuscript. XZ coordinated and directed the project. All authors contributed to the article and approved the submitted version.
