# Supplementary figures and images for: Donor derived hematopoietic stem cell niche transplantation facilitates mixed chimerism mediated donor specific tolerance
Source: Front Immunol. 2023 Feb 16;14:1093302. doi: 10.3389/fimmu.2023.1093302 (PMC9978155; doi:10.3389/fimmu.2023.1093302)

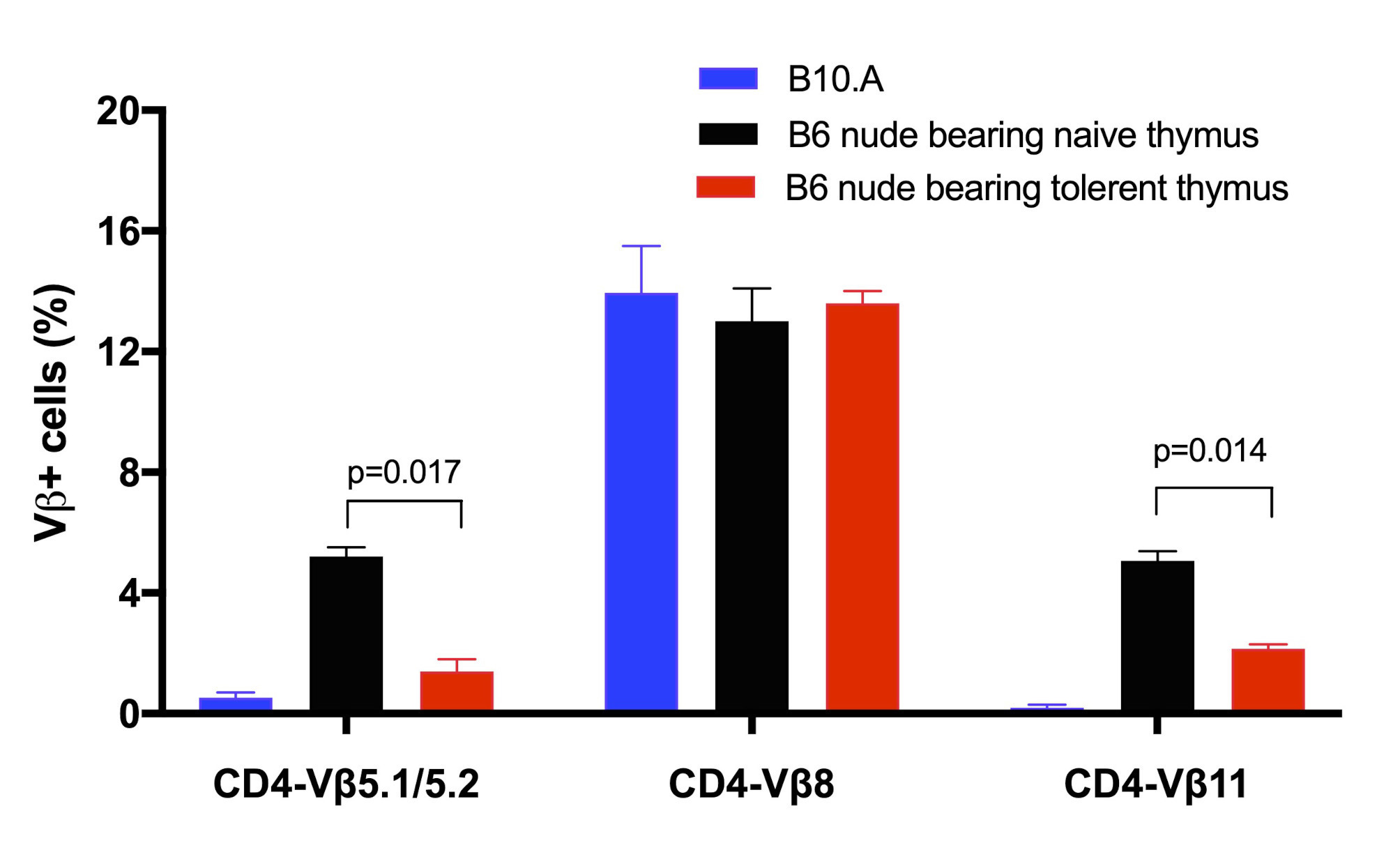

Supplement: Supplementary file 1 [file Image_1.jpeg]
